# Nonmotor symptoms in primary adult‐onset cervical dystonia and blepharospasm

**DOI:** 10.1002/brb3.592

**Published:** 2016-12-18

**Authors:** Jing Yang, Na Shao, Wei Song, Qianqian Wei, Ruwei Ou, Ying Wu, Hui‐Fang Shang

**Affiliations:** ^1^ Department of NeurologyWest China HospitalSichuan UniversityChengduSichuanChina

**Keywords:** anxiety, cognition, depression, primary focal dystonia, quality of sleep

## Abstract

**Background:**

The nature and frequency of nonmotor symptoms in primary adult‐onset cervical dystonia (CD) and blepharospasm (BSP) patients in Chinese populations remain unknown.

**Methods:**

Hamilton's Depression Scale (HAMD), Hamilton's Anxiety Scale (HAMA), Addenbrooke's Cognitive Examination Revised (ACE‐R), Pittsburgh Sleep Quality Index and Epworth Sleepiness Scale were used to evaluate NMS in 120 patients with primary focal adult‐onset dystonia (60 with BSP and 60 with CD) and 60 age‐, sex‐, and education level‐ matched healthy controls (HCs). Motor symptoms of BSP and CD patients were evaluated by Jankovic rating scale and Toronto Western Spasmodic Torticollis Rating Scale‐severity scale separately.

**Results:**

Twenty patients had depression, and 29 patients had anxiety. The mean HAMD and HAMA scores were significantly higher in patient groups. Thirty‐six patients had cognitive decline based on the cut‐off score of 75. The total score and scores of each domain of ACE‐R were significantly lower in patient groups than that in HCs. Quality of sleep was impaired in patient groups, and patients with CD had worse quality of sleep than patients with BSP. Thirty‐three BSP patients and 43 CD patients suffered from sleep disorder separately. The frequency of excessive daytime sleepiness did not differ between patients and HCs. No significant correlation was found between NMS and motor severity in the two forms of dystonia.

**Conclusions:**

Current study suggests that NMS are prevalent in Chinese CD and BSP patients, and the motor severity of dystonia did not contribute to the severity of nonmotor symptoms. Assessment of nonmotor symptoms should be considered in clinical management of focal dystonia

## 1 Introduction

Dystonia is a movement disorder characterized by sustained or intermittent muscle contractions causing abnormal, often repetitive, movements, postures, or both. Dystonic movements are typically patterned, twisting, and may be tremulous. Dystonia is often initiated or worsened by voluntary action and associated with overflow muscle activation (Albanese et al., 2013). According to the involvement of body region, dystonia can be classified into focal, segmental, multifocal, hemidystonia, and generalized dystonia (Albanese et al., 2013). Cervical dystonia (CD) and blepharospasm (BSP) are the most common forms of focal dystonia, with a prevalence of 4.98 per 100 000 and 4.24 per 100 000 separately (Steeves, Day, Dykeman, Jette, & Pringsheim, 2012).

Recently, increasing evidence has suggested that patients with dystonia suffered from not only dystonic movement and/or postures, but also a series of nonmotor symptoms (NMS) such as depression, anxiety, low self‐esteem, and sleep disorders (Avanzino et al., 2010; Ben‐Shlomo, Camfield, & Warner, 2002; Eichenseer, Stebbins, & Comella, 2014; Fabbrini et al., 2010; Gundel et al., 2003; Romano et al., 2014). Sleep disorders were reported commonly in patients with dystonia (Avanzino et al., 2010; Eichenseer et al., 2014). Avanzino et al. (2010) found BSP patients suffered from impairment in sleep duration, latency, and efficiency, while they did not find significant sleep disturbances in patients with CD. However, a small sample prospective study found that the quality of sleep was significantly impaired in patients with CD, and that the quality of sleep or daytime somnolence was not improved despite the improvement of the motor severity of CD patients (Eichenseer et al., 2014). Romano et al. (2014) reported patients with cranial‐cervical dystonia might have impairment in specific cognitive domains which are related to working memory, processing speed and short‐term memory. However, cranial dystonia and cervical dystonia were not separately analyzed in the above study. Aleman reported BSP patients performed poorly on a variety of cognitive tasks requiring prefrontal or parietal function (Aleman, de Erausquin, & Micheli, 2009), but whether CD and BSP patients both suffered similar cognitive impairments remains unknown. Furthermore, the relationship between clinical features and NMS in each specific primary focal dystonia remains unknown.

Several studies have provided convincing evidence that NMS are important predictors for quality of life in dystonia (Ben‐Shlomo et al., 2002; Eichenseer et al., 2014; Page, Butler, & Jahanshahi, 2007; Pekmezovic et al., 2009). A study using “36‐item short‐form healthy survey (SF‐36)” scale found anxiety and depression are the strongest predictors for quality of life in CD (Ben‐Shlomo et al., 2002), which was supported by the studies on patients with various forms of primary dystonia (Page et al., 2007; Pekmezovic et al., 2009). Quality of life and the contribution of nonmotor symptoms to quality of life were also recently assessed in genetic forms of dystonia (Bruggemann et al., 2014). Therefore, better understanding the NMS of dystonia is helpful for improving the quality of life of patients with dystonia.

No studies on NMS of primary dystonia in Chinese population are available. Therefore, we conducted the current cross‐sectional study to explore the features of NMS and analyze the relationship between NMS and clinical features in CD and BSP patients in China. Meanwhile, we also explored whether the two forms of dystonia have different patterns of NMS that allow to distinction between them.

## 2 Patients and Methods

### 2.1 Participants

The study comprised 120 primary focal dystonia patients, including 60 CD patients and 60 BSP patients who were admitted to the Department of Neurology, West China Hospital, Sichuan University between September 2013 and January 2015. The diagnoses were made by a neurologist with long‐standing experience in movement disorders (HF Shang) based on the published criteria (Fahn, Marsden, & Calne, 1987; Hallett, Evinger, Jankovic, & Stacy, 2008). Known causes of secondary dystonia were excluded on the basis of medical and drug histories, neurological examination, laboratory investigation and abnormal findings on conventional MRI. None of the patients had other neurological abnormalities or family history of movement disorders. The demographic and clinical features including age, age of onset, disease duration, treatment regimen, and botulinum toxin treatment, were collected by using a standard questionnaire during face‐to‐face interviews. Sixty healthy controls (HCs) matched for age, sex, and education years were included. This study was approved by the ethical committee of West China Hospital of Sichuan University. All participants were informed about the purpose of the study, and their informed consents were obtained.

### 2.2 Assessment of the motor symptoms and NMS

Motor symptom of CD was evaluated by Toronto Western Spasmodic Torticollis Rating Scale (TWSTRS)‐ severity scale (Comella et al., 1997). Motor symptom of BSP was evaluated by using the Jankovic rating scale (JRS) (Jankovic, Comella, Hanschmann, & Grafe, 2011). The severity of depression was evaluated by the 24‐items Hamilton's Depression Scale (HAMD) (Hamilton, 1960). A score of HAMD more than 20 indicates depression. The severity of anxiety was assessed by 14‐items Hamilton's anxiety scale (HAMA) (Hamilton, 1959) and a score more than 14 indicates anxiety. The quality of sleep was measured by Pittsburgh Sleep Quality Index (PSQI) (Buysse, Reynolds, Monk, Berman, & Kupfer, 1989), which is a self‐rated questionnaire that assesses seven domains of sleep. The maximum score of PSQI is 21, and the score greater than 5 indicates poor sleep quality. Excessive daytime somnolence was assessed by Epworth Sleepiness Scale (ESS) (Johns, 1991), which is a validated measure to rate the chances of falling asleep during a variety of situations. A score of 10 or more is considered to have excessive daytime sleepiness. Cognitive function was evaluated by using the Chinese version of Addenbrooke's Cognitive Examination Revised (ACE‐R) (Mioshi, Dawson, Mitchell, Arnold, & Hodges, 2006), which contains five domains: attention/orientation (18 points), memory (26 points), fluency (14 points), language (26 points), and visuospatial (16 points). The maximum score of ACE‐R is 100, with higher score representing better cognition function. Cognitive impairment was defined as a total score less than 1.5 standard deviations of the controls’ mean on the ACE‐R (Nazem et al., 2009).

### 2.3 Statistical analysis

All data were analyzed using SPSS 16.0. For the statistical analysis, data were entered in two different analyses comparison: (1) the total patients group including CD and BSP patients compared with matched HCs; (2) CD patients compared with BSP patients. Comparisons of continuous variables between groups were analyzed using the Student's *t*‐tests when the variables met the normal distribution or Wilcoxon rank sum tests when the variables were not normally distributed. Chi‐square test was used for the comparison of categorical variables. A linear regression model was performed to explore the associations between the HAMD scores, HAMA scores and motor severity rating scores separately. Multiple liner regression analysis was used to examine the association between cognitive performance and clinical variables including severity of motor symptoms, age, and education years, as well as the association between sleep quality and clinical variables including motor severity rating scores, anxiety, and depression. In addition, considering the potential confounding effects of depression and anxiety on the sleep quality, an Analysis of Covariance (ANCOVA) was used to study the differences in PSQI and ESS scores between patients and HCs groups. As the mean age and education years were different between CD and BSP patients groups, we also compared the differences in every NMS between CD group and BSP group after adjustment for age and education years by using ANCOVA. A *p *<* *.05 was considered to be statistically significant.

## 3 Results

### 3.1 Demographic and clinical features of subjects

Main demographic and clinical features of the subjects are summarized in Table 1. Among the patients with CD, 17 patients were receiving treatment for their dystonia at the evaluation date, such as trihexyphenidyl and clonazepam, two patients received botulinum toxin injection 4 months and 1 month ago separately, 14 patients had received treatment for their dystonia several months ago, and the rest patients did not receive any treatment before. For patients with BSP, 21 patients were receiving treatment for their dystonia, two patients received botulinum toxin injection before (both received more than 6 months before), 10 had received treatment for their dystonia before, and the rest patients never received treatment.

**Table 1:** Characteristics of subjects

	Dystonia patients			HCs	*p* value	
	Total	CD	BSP		Total vs. HCs	CD vs. BSP
Number (M/F)	120 (47/73)	60 (25/35)	60 (22/38)	60 (30/30)	.110	.709
Mean age (SD)	47.58 (14.42)	40.23 (13.30)	54.94 (11.51)	47.87 (14.18)	.901	<.001
Age of onset (SD)	45.56 (14.74)	38.46 (14.56)	52.67 (11.31)	–	–	<.001
Disease duration (year)	2.13 (3.66)	2.65 (4.32)	3.20 (3.85)	–	–	.466
Education years	9.91 (3.64)	10.17 (3.68)	9.65 (3.62)	11.13 (4.83)	.087	.440
Depression (yes/no)	20/100	12/48	8/52	0/60	<.001	.463
Anxiety (yes/no)	29/91	17/43	12/48	2/58	<.001	.394
Cognitive disorder (yes/no)	36/84	15/45	21/39	5/55	.001	.319
Sleep disorder (yes/no)	76/44	43/17	33/27	15/45	<.001	.088
Excessive daytime Sleepiness (yes/no)	27/93	12/48	15/45	14/46	1	.662

CD, cervical dystonia; BSP, blepharospasm; HCs, healthy controls; F, female; M, male; SD, standard deviation.

### 3.2 Evaluation of motor symptoms

The mean JRS score in BSP patients was 5.55 ± 1.81. Motor symptom of 46 BSP patients (76.7%) became worse under emotional stress and of 51 BSP patients (85%) was improved by “sensory trick” by touching bilateral geison and cheeks with fingers. The mean TWSTRS motor severity score of CD patients was 15.98 ± 5.17. Motor symptom of 27 CD patients (45%) became worse under emotional stress and of 58 patients (96.7%) was improved by “sensory trick” by touching the back neck.

### 3.3 Evaluation of depression and anxiety

For all included dystonia patients, 20 patients (16.7%) had depression and 29 (24.2%) had anxiety. The mean HAMD and HAMA scores in patients group were significantly higher than that in HCs groups (*p *<* *.01, Table 2). Two healthy subjects suffered anxiety. Among the CD patients, six male and six female patients (20%) suffered depression, eight male and nine female patients (28.3%) suffered anxiety. Among the BSP patients, five female and three male patients (13.3%) had depression, six male and six female patients (20.0%) had anxiety. The mean HAMD and HAMA scores were not significantly different between CD patients and BSP patients groups (Table 2). There were no significant differences in disease durations between patients with and without depression or anxiety. According to the linear regression analysis, there were no significant correlations between motor symptoms and HAMD scores or HAMA scores.

**Table 2:** Prevalence of each domain of NMS of included BSP, CD patients and matched HC

	Total (SD)	CD (SD)	BSP (SD)	HCs (SD)	*p* value	
					Total vs. HCs	CD vs. BSP
HAMD	11.92 (8.10)	13.17 (8.24)	10.68 (7.83)	2.47 (3.79)	<.001	.093
HAMA	7.07 (5.52)	7.58 (5.75)	6.55 (5.28)	2.18 (3.63)	<.001	.307
ACE‐R (total)	80.32 (12.26)	81.93 (10.79)	78.72 (13.47)	88.55 (9.00)	<.001	.151
Orientation	16.71 (1.65)	16.67 (1.60)	16.75 (1.71)	17.63 (0.84)	<.001	.784
memory	21.60 (4.19)	21.98 (3.66)	21.22 (4.66)	23.85 (2.31)	<.001	.318
fluency	9.37 (2.31)	9.40 (2.07)	9.33 (2.54)	10.40 (2.35)	.005	.875
language	19.10 (5.09)	19.90 (4.76)	18.30 (5.33)	21.95 (3.84)	<.001	.085
visuospatial	13.62 (3.07)	13.97 (2.58)	13.28 (3.47)	14.70 (2.11)	.007	.224
PSQI	7.28 (4.32)	8.22 (4.58)	6.35 (3.87)	3.85 (3.23)	<.001	.017
ESS	7.17 (4.74)	6.78 (4.67)	7.55 (4.82)	6.35 (4.79)	.279	.378

NMS, non‐motor symptoms; CD, cervical dystonia; BSP, blepharospasm; HC, healthy control; SD, standard deviation; ACE‐R, Addenbrooke's Cognitive Examination Revised; PSQI, Pittsburgh Sleep Quality Index; ESS, Epworth Sleepiness Scale.

### 3.4 Cognitive assessment

The mean score of ACE‐R of healthy controls was 88.55. The mean total ACE‐R scores (80.32) and the scores of each domain in patients group were significantly lower than that in HCs (Table 2). In the current study, cognitive impairment was defined as a total score less than 75. Thirty‐six patients including 15 CD patients (25%) and 21 BSP patients (35%) suffered from cognitive deficits. There were no significant differences in the total or the scores of each domain of ACE‐R between the BSP and CD patients groups (Table 2). Multiple liner regression analysis showed that ACE‐R score was positively correlated with education level (β = 2.322, *p *< .001 for BSP patients; β = 1.633, *p *< .001 patients groups) but not correlated with the severity of motor symptoms in BSP or CD patients, and negatively correlated with age in BSP patients (β = −0.405, *p *= .005).

### 3.5 Sleep quality and excessive daytime sleepiness assessment

PSQI scores were significantly higher in patients group than in HCs. CD patients had worse sleep quality than BSP patients (*p *=* *.017). Forty‐three patients with CD (71.7%) were defined as poor sleepers by PSQI >5, and 12 CD patients (20%) were defined as excessive daytime sleepers. Thirty‐three patients with BSP (55%) were defined as poor sleepers, and 15 BSP patients (25%) as excessive daytime sleepers. There were no significant differences in disease durations between patients with and without poor sleep or daytime somnolence. The frequency of excessive daytime sleepiness measured by ESS was not significantly different between patients group and HCs. However, after adjustment for depression and anxiety, the ANCOVA analysis found no significant differences in PSQI or ESS scores between patients and HCs (*p *=* *.416 and *p *=* *.782, separately). Multiple liner regression analysis indicated that there were positive correlations between depression and PSQI scores in both CD (β = 0.292, *p *< .001) and BSP patients (β = 0.353, *p *< .001), while no correlation was found between PSQI and HAMA or motor symptoms.

## 4 Discussion

To the best of our knowledge, our study is the first to assess numerous NMS including mood disorders, cognitive impairment and sleep disorder of Chinese patients with BSP and CD. We found that NMS including depression, anxiety, worse quality of sleep and cognitive dysfunction are common in Chinese craniocervical focal dystonia patients. Motor severity did not contribute to the severity of each NMS neither in CD nor in BSP. CD patients suffered from worse quality of sleep than BSP patients, however, no differences were found in the severity of depression, anxiety, and cognitive dysfunction between CD patients and BSP patients.

High frequency of depression and anxiety observed in our BSP and CD patients resembles the results of some other studies (Fabbrini et al., 2010; Gundel et al., 2003; Lencer et al., 2009). Muller et al. (2002) found depression of some BSP and CD patients did not improve correspondingly after significant relief of motor symptom and pain by botulinum toxin A treatment, which supported our findings that no significant correlation was found between depression and motor severity. The observation that more than half of CD patients developed depression before the onset of motor symptom (Wenzel et al., 1998), challenged the traditional view that mood disorders were secondary to motor disability in dystonia. Although the pathogenesis of depression and anxiety in dystonia patients remains unclear, neurophysiological researches reported that cortico‐striatal‐thalamo‐cortical circuits contributed to not only in motor symptoms, but also in psychiatric symptoms (Stamelou, Edwards, Hallett, & Bhatia, 2012; Vuilleumier et al., 2001). Meanwhile, neuroimaging studies of dystonia also found structural abnormalities in brain regions including cingulate cortex, precuneus, prefrontal area, thalamus, which are involved in emotion regulation (Gong & He, 2015; Sexton, Mackay, & Ebmeier, 2013). Genetic factor also contributes to psychiatric disorders in dystonia patients. For example, early‐onset recurrent major depression was found to be associated with the DYT1 GAG mutation and this association was independent of motor manifestations of dystonia (Heiman et al., 2004). Patients with myoclonus dystonia suffered more frequently from obsessive‐compulsive disorder and alcohol abuse, which might be partly caused by the mutation of epsilon‐sarcoglycan (*SGCE)* gene (Peall et al., 2013). Our results together with these findings indicate that mood disorders may be primary phenotypic components of this disorder. More longitudinal studies are needed to prove the relationship between mood disorders and dystonia in the future, and to determine whether psychological intervention may help patients to modify their perceptions of this disease.

In the current study, we found poor cognitive performance in CD and BSP patients by using ACE‐R. Attention deficit was reported by recent studies of patients with different types of dystonia, including primary generalized dystonia and focal dystonia (Allam, Frank, Pereira, & Tomaz, 2007; Scott et al., 2003). Attention deficit may indicate the disruption of the dorsolateral‐prefrontal loop, which involves the striatum, thalamus and prefrontal cortex, regions showing altered functional activity and microstructural abnormalities in dystonia (Yang et al., 2013; Zoons, Booij, Nederveen, Dijk, & Tijssen, 2011). Deficits in visuospatial functioning have also been reported in CD and BSP patients and were not correlated with severity or duration of dystonia, which may reflect dysfunction in striatal‐frontal circuits (Aleman et al., 2009; Hinse et al., 1996). Mental control and visual reproduction impairment in CD was reported by Romano et al. (2014). The deficit in verbal fluency was also reported in another group of primary dystonia patients with different types of body distribution. Neuroimaging studies have revealed altered functional activity and glucose metabolism in frontal and temporal lobes, regions are associated with verbal fluency, which may provide the neuroanatomical basis for deficits in verbal fluency (Kerrison et al., 2003; Schmidt et al., 2003). The lack of correlation between cognitive performance and severity of motor symptoms indicate that cognitive decline may be a clinical expression of dystonia, which was supported by the broad cortical involvements with either the disruption occurring at the frontal‐subcortical loops involving the basal ganglia, or the dysfunction of other nonmotor regions including cingulate, occipital lobe, parietal, or temporal lobe (Yang et al., 2013; Zoons et al., 2011).

CD and BSP patients suffered worse quality of sleep when compared to HCs, and CD patients suffered worse quality of sleep than BSP patients. Lower sleep quality in CD compared to BSP might be due to pain, which is common in CD but absent in BSP. Lack of correlation between PSQI and severity of dystonia in both CD and BSP patients suggested that insomnia might be a comorbidity disorder in patients with BSP or CD, which partly resemble the results of Avanzino et al. (2010). After ANCOVA analysis to avoid the influence of anxiety and depression, insomnia showed tight connection with depression, but the causal relationship between quality of sleep and depression remains unknown (Lobbezoo, Thu Thon, Remillard, Montplaisir, & Lavigne, 1996). Besides PSQI, we used ESS to evaluate daytime sleepiness. It's interesting to find that the impairment of sleep quality did not have impact on daytime sleepiness, which was similar with another study (Avanzino et al., 2010). Overall, insomnia is a feature of primary cranial and cervical dystonia.

This study has some limitations. On the one hand, this is a cross‐sectional study, which cannot indicate causal relationship between NMS and motor symptoms. On the other hand, some other NMS such as fatigue, pain, obsessive‐compulsive disorder are not considered in the present study. In addition, our patients did not receive genetic screening, so we could not explore the possible effects of gene mutation on clinical symptoms.

In conclusions, NMS are prevalent in Chinese cranial or cervical focal dystonia patients. The depression, anxiety, cognitive disorders, and sleep disorders are predominant in CD and BSP patients, and the severity of NMS reported in the present study does not have significantly correlation with motor severity of dystonia.

## Conflict of Interest

The authors declare that they have no conflict of interest.
